# Evasion and Immuno-Endocrine Regulation in Parasite Infection: Two Sides of the Same Coin in Chagas Disease?

**DOI:** 10.3389/fmicb.2016.00704

**Published:** 2016-05-23

**Authors:** Alexandre Morrot, Silvina R. Villar, Florencia B. González, Ana R. Pérez

**Affiliations:** ^1^Institute of Microbiology, Federal University of Rio de JaneiroRio de Janeiro, Brazil; ^2^Institute of Clinical and Experimental Immunology of Rosario, CONICET, National University of RosarioRosario, Argentina; ^3^Faculty of Medical Sciences, National University of RosarioRosario, Argentina

**Keywords:** evasion strategies, persistence, Chagas disease, immunoendocrine, thymus, virulence factors

## Abstract

Chagas disease is a serious illness caused by the protozoan parasite *Trypanosoma cruzi*. Nearly 30% of chronically infected people develop cardiac, digestive, or mixed alterations, suggesting a broad range of host-parasite interactions that finally impact upon chronic disease outcome. The ability of *T. cruzi* to persist and cause pathology seems to depend on diverse factors like *T. cruzi* strains, the infective load and the route of infection, presence of virulence factors, the parasite capacity to avoid protective immune response, the strength and type of host defense mechanisms and the genetic background of the host. The host-parasite interaction is subject to a constant neuro-endocrine regulation that is thought to influence the adaptive immune system, and as the infection proceeds it can lead to a broad range of outcomes, ranging from pathogen elimination to its continued persistence in the host. In this context, *T. cruzi* evasion strategies and host defense mechanisms can be envisioned as two sides of the same coin, influencing parasite persistence and different outcomes observed in Chagas disease. Understanding how *T. cruzi* evade host's innate and adaptive immune response will provide important clues to better dissect mechanisms underlying the pathophysiology of Chagas disease.

## Introduction

*Trypanosoma cruzi* is a protozoan parasite that causes Chagas disease (WHO, 2015). Nearly 30% of chronically infected people develop cardiomyopathy, megacolon, and megaesophagus or a mixed of these alterations, suggesting a broad range of host-parasite interactions that finally impact upon chronic disease outcome (Rassi et al., 2010). Different and not mutually exclusive hypotheses have been considered for the pathogenesis of chronic Chagas disease, including autoimmunity by molecular mimicry, microvascular (Ramos and Rossi, 1999), and autonomic dysfunction (Dávila et al., 2004), and tissue damage by parasite persistence (Gironès et al., 2005; Gutierrez et al., 2009; Cunha-Neto et al., 2011). The parasite persistence hypothesis predicts a chronic inflammatory reactivity as result of a failure in parasite burden control, thus promoting the development of disease pathology (Tarleton, 2001). In addition, a subpatent parasite-induced cell lysis as consequence of amastigote differentiation into trypomastigotes (Bonney and Engman, 2008) might fuel inflammation. The presence of parasites (Añez et al., 1999; Buckner et al., 1999) or their products, such as DNA, in blood and myocardium of chronic infected hosts is well documented (Añez et al., 1999; Zhang and Tarleton, 1999; Salomone et al., 2000; Elias et al., 2003). *T. cruzi* reactivation in HIV co-infected, transplanted or immunocompromised chronic chagasic patients provides convincing evidence of parasite persistence (Tarleton, 2001; Andrade et al., 2014), reinforcing the view that disease pathology and its severity are directly related to *T. cruzi* presence within the affected tissue (Tarleton, 2001). In this review, we examined the complexity of cellular, molecular and physiologic factors involved in *T. cruzi* evasion and persistence in the light of current data.

## Parasite evasion involve direct host immune regulation and latency establishment

**Figure 1:**
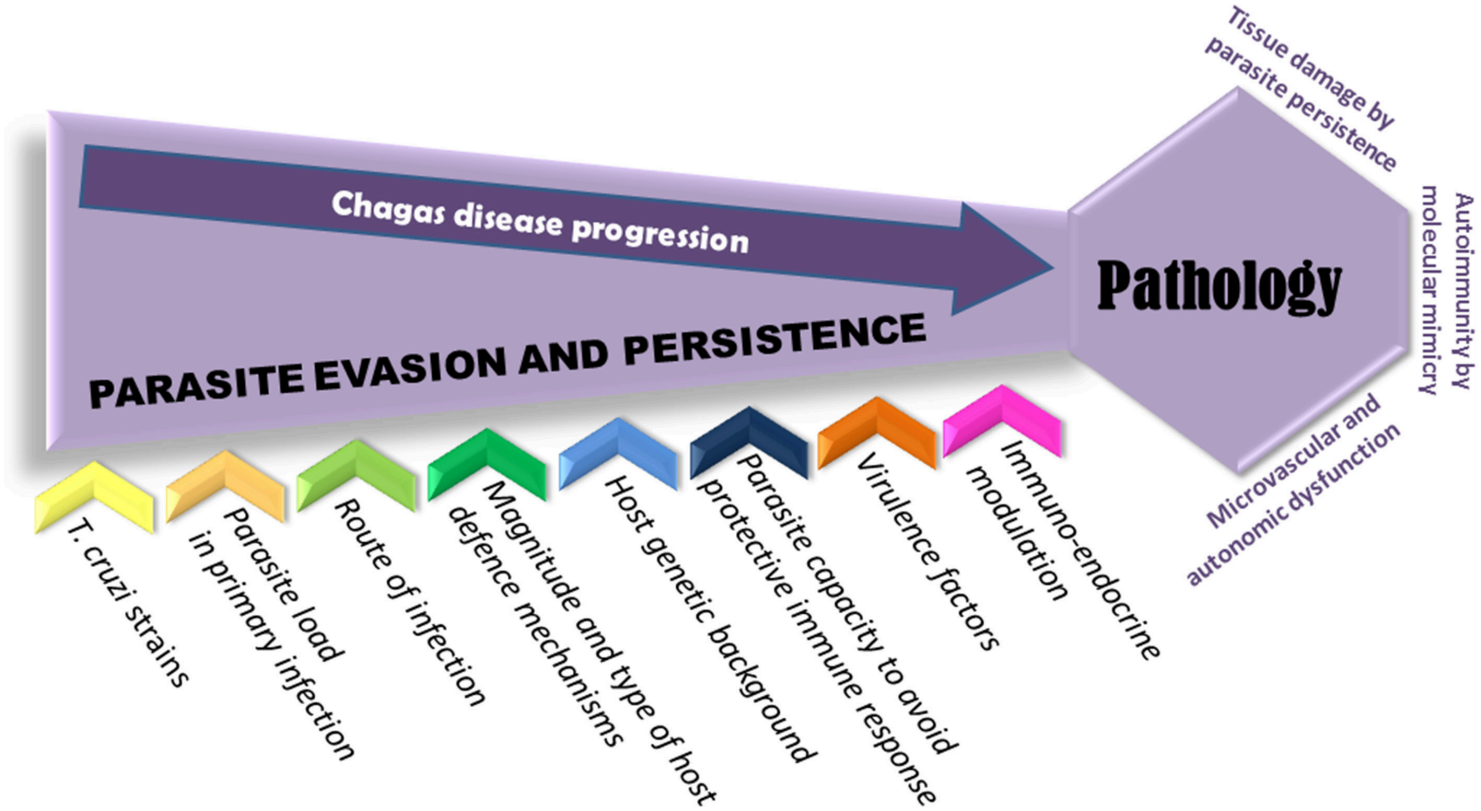
**Pathogenesis of *Trypanosoma cruzi* infection**. Several hypotheses have been considered for the pathogenesis of chronic Chagas disease, comprising tissue damage by parasite persistence, autoimmunity, microvascular injury, and autonomic dysfunction. Since diverse factors are involved in parasite evasion and persistence, most of all may influence the infection outcome and the development of pathology in almost 30% of infected individuals. The ability of *T. cruzi* to evade immune system seems to depend on diverse factors like *T. cruzi* strains, the infective load and the route of infection and the presence of virulence factors; but also can be determined by the type and strength of host defense mechanisms and the genetic background of the host.

**Figure 2:**
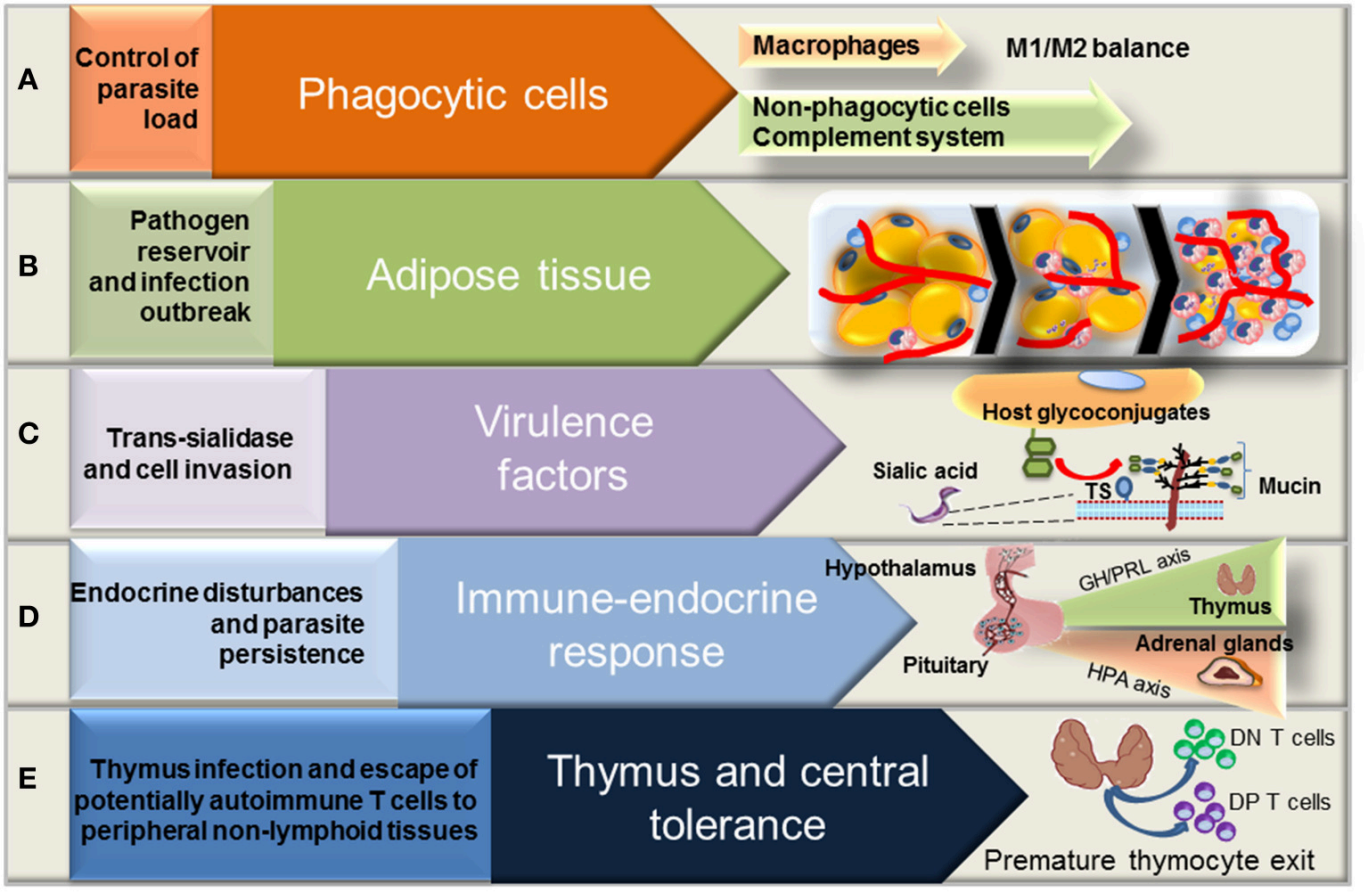
**Aspects of *Trypanosoma cruzi* evasion and persistence in the vertebrate host**. *T. cruzi* parasites develop different strategies to evade the host defenses and establish a persistent infection. *T. cruzi* parasites evade the host innate immune responses associated with macrophage and complement system **(A)**. The trans-sialidase (TS), a *T. cruzi*-derived virulence factor, can also overcome the host resistance responses to optimize the invasion and parasite persistence in chronic infection **(B)**. The development of anti-parasite immune response is coupled with the activation of neuroendocrine axes that may affect the course of disease **(C)**. Adipose tissue can be considered as a parasite reservoir and may contribute to the establishment of persistent infections, playing a major role in *T. cruzi* burst during immunosuppression periods **(D)**. The recognition of *T. cruzi*-derived antigens in the thymus may restrict the central tolerance to parasite infection, while the release of immature and potentially autoimmune T cells to the peripheral non-lymphoid tissues may be related with disease pathology in the chronic phase **(E)**.

*T. cruzi* has a complex biological cycle involving mammals and insect vectors. The strategies that *T. cruzi* employs to guarantee its long-term survival within mammalian hosts include evasion from phagolysosome, expression of virulence factors, direct immunomodulation and the establishment of latency sites (Figure 1). Trypomastigotes can invade nucleated cells through different mechanisms depending on whether the target cell is phagocytic or nonphagocytic (Figure 2A; Romano et al., 2012). Macrophages are the most important innate effector cells in the fight against *T. cruzi*, but when subverted in the infection they can be also exploited by the parasite as its primary niche, thus avoiding cell-mediated immunity. Protective classically activated (M1) macrophages are activated by IFN-γ, increasing the expression of nitric oxide synthase (iNOS) and nitric oxide (NO) production favoring the parasite killing. In contrast, parasite clearance is prevented when macrophages acquire an alternatively activated (M2) phenotype, with reduced NO production thus increasing the parasite persistence (Sizirensen et al., 1994; Desjardins and Descoteaux, 1997; Paulnock and Coller, 2001; Stempin et al., 2002; Martinez and Gordon, 2014). Unlike other parasites that prevent phagolysosome maturation (David Sibley, 2011), *T. cruzi* evades macrophage microbicidal activity by escaping from phagolysosome to cytoplasm, an event that is mediated by the cytolitic activity of parasite's C9 cross-reactive protein (Tc-Tox; Andrews et al., 1990; Bogdan and Röllinghoff, 1999). Once inside the cytoplasm, *T. cruzi* parasites promote STAT1 dephosphorylation, thus interfering with the transcription of IL-12 and TNF-α (De Diego et al., 1997) that ultimately abrogate IFN-γ-mediated microbicidal responses (Gazzinelli et al., 1992; Stahl et al., 2014). In addition, parasite-derived proteases shutdown IL-12 expression by interrupting the NF-κB signaling pathway (Doyle et al., 2011). Furthermore, *T. cruzi* stimulate the secretion of anti-inflammatory cytokines such as IL-10 and TGF-β that impair the development of protective immune responses hence favoring the spread of infection and parasite persistence in the host (Silva et al., 1991; Hunter et al., 1997; Freire-de-Lima et al., 2000). *T. cruzi* can also disrupt the classical and alternative complement pathways: parasite CRP and T-DAF proteins bind to C3b and C4b fragments, inhibiting the assembly of C3 and C5 convertase on the parasite membrane (Joiner et al., 1986; Norris et al., 1991; Tambourgi et al., 1993; Zambrano-Villa et al., 2002).

The acute phase of infection is marked by a transient state of immunosuppression (Liew et al., 1987; Kierszenbaum et al., 1999, 2002; Van Overtvelt et al., 2002; Alcaide and Fresno, 2004; Gutierrez et al., 2009; Padilla et al., 2009; DosReis, 2011; Oladiran and Belosevic, 2012; Pinazo et al., 2013) involving, among other things, a strong polyclonal B cell stimulation which restricts the development of antigen-specific lymphocytes, promoting apoptosis and cell cycle arrest (Ortiz-Ortiz et al., 1980; Maleckar and Kierszenbaum, 1983; Zuñiga et al., 2000). In fact *T. cruzi* provides a good example of such immunosuppression strategy: T cells from infected mice respond poorly to mitogens (Kierszenbaum et al., 1999, 2002; Alcaide and Fresno, 2004) and they also undergo enhanced apoptosis upon stimulation of T cell receptor (TCR), increasing the unresponsiveness of host immunity (Abrahamsohn and Coffman, 1995; Martins et al., 1998; Nunes et al., 1998). Studies have supported that IL-2 deficiency is one of the hallmarks of the disease-induced T cell immunosuppression (Abrahamsohn and Coffman, 1995). The T cell unresponsiveness in Chagas disease is also the result of a direct downregulation of IL-2 receptor by the parasite glycoprotein AGC10 (Kierszenbaum et al., 1999). Recently, a novel immunosuppressive mechanism was described, which implies the IFN-γ-dependent NO secretion by immature myeloid cells (Goñi et al., 2002).

## Adipose tissue acts as a reservoir of *Trypanosoma cruzi*

Another adaptive strategy displayed by *T. cruzi* parasites to optimize its persistence in the host consists in targeting tissues with particular characteristics. Some studies have shown that adipose tissue (AT) might serve as a reservoir where parasite could persist in a latent state to avoid the host-defense mechanisms, acting as a possible site of reactivation, similarly to that observed for other intracellular pathogens (Figure 2B; Neyrolles et al., 2006; Bechah et al., 2010, 2014). Adipocytes could harbor a significant number of parasites even in the chronic phase of infection (Combs et al., 2005). Recently, more robust evidence that AT can act as a reservoir of *T. cruzi* have arisen from experiments in which infected mice were subsequently treated with an anti-parasitic drug and afterwards immunosuppressed. Intriguingly, in a significant number of animals, the AT was the major site of parasite recrudescence (Fortes Francisco et al., 2015). Moreover, studies carried out in patients with chronic chagasic cardiomyopathy have revealed the presence of parasite-derived DNA in AT (Ferreira et al., 2011). In this sense, AT may be a reservoir from which reactivation of infection may occur, especially during periods of immunosuppression, as observed in chagasic patients co-infected with HIV, transplanted or undergoing immunosuppressive therapies (Almeida et al., 1996; Sartori et al., 1998; Campos et al., 2008; Cordova et al., 2008; Pinazo et al., 2013). Moreover, in HIV co-infected chagasic patients, periods of lipoatrophy may result in the release of parasites into the circulation (Ferreira et al., 2011). It remains to be investigated why *T. cruzi* persists in the AT. Possible reasons could be the special metabolic conditions that *T. cruzi* finds inside the adipocyte and the slow turnover of these cells. After infection, there is an influx of inflammatory cells to AT, accompanied by an *in situ* upregulation of both TNF-α and IL-6, concomitantly to a diminution of adipocytokine levels (Desjardins and Descoteaux, 1997; Chandrasekar et al., 2000; Manarin et al., 2013). Moreover, some studies suggested that metabolic alterations induced by *T. cruzi* persistence in AT may increase the risk of diabetes, metabolic syndrome and cardiovascular disease (Chandrasekar et al., 2000; Nagajyothi et al., 2009; Manarin et al., 2013).

## *Trypanosoma cruzi* virulence factors overcome the host resistance response to establish persistent infections

The ability of *T. cruzi* parasites to persist and cause pathology partially depends on factors such as the parasite strain, the genetic background of the host (Andrade et al., 2002; Savino et al., 2007) and the route of infection (Barreto-de-Albuquerque et al., 2015). *T. cruzi* species display a broad range of biological, biochemical, molecular and genetic characteristics, being grouped in six discrete typing units (DTUs; Macedo et al., 2004; Zingales et al., 2009). The parasite immune modulatory effects seems to be strain-dependent, a feature that might influence parasite-host interactions (Lauria-Pires, 1996; Briones et al., 1999). Different parasite strains coexist dynamically in natural reservoirs and combinations of them have been found in triatomine bugs from domestic and peridomestic areas (Noireau et al., 2009), as well as in infected patients (Cura et al., 2015; Monje-Rumi et al., 2015).

The damping of host immune response during the acute phase of Chagas disease is partially caused by *T. cruzi*-derived virulence factors (Figure 2C; DosReis et al., 2005; Gutierrez et al., 2009; Nagajyothi et al., 2012). One of the hallmarks of parasite's cell membrane composition is the presence of mucin-like molecules presenting sialic acid residues attached to their terminal β-galactosyl residues. These residues are transferred from host glycoconjugates by the action of parasite trans-sialidase (Schenkman et al., 1991, 1994; Previato et al., 1995; Eugenia Giorgi and De Lederkremer, 2011). Parasite mucins are encoded by more than 800 genes comprising ~1% of the parasite genome, represented as O-glycosylated Thr/Ser/Pro-rich glycoproteins (Di Noia et al., 1995; Buscaglia et al., 2006; Mendonça-Previato et al., 2013). The *T. cruzi*-derived mucin molecules are determinant in the host-parasite interplay, since they mediate processes related to invasion of the vertebrate cells as well as subvert the host immune system. The sialylated forms of mucin-like molecules protect the parasite antigenic determinants from host humoral responses, avoiding the humoral attack mediated by anti-galactosyl antibodies and complement factor B (Kipnis et al., 1981; Joiner et al., 1986; Gazzinelli et al., 1991; Pereira-Chioccola et al., 2000). Moreover, it has been shown that once sialylated, mucin-like molecules are able to impair the host dendritic cell function through inhibition of the IL-12 expression (Erdmann et al., 2009), possibly at transcriptional level as described for IL-2 gene (Kierszenbaum et al., 1999, 2002). Furthermore, the parasite sialoglycoproteins are able to inhibit tyrosine phosphorylation of the adapter protein SLP-76 and tyrosine kinase ZAP-70, both involved in the early events of T cell activation (Alcaide and Fresno, 2004).

Recently, it has been shown that *in vivo* exposure to *T. cruzi* mucins enhances the host susceptibility, as seen by the increased parasitemia and heart tissue damage. These effects were associated with a reduction in Th1 and Th2 cytokine production, together with decreased levels in the frequency of IFN-γ producing CD4^+^ T cells in the spleen of mice treated with parasite mucins in comparison with untreated controls (Nunes et al., 2013). Interestingly, it has been shown that the binding of acid-binding Ig-like lectin Siglec-E (CD33) by *T. cruzi* mucins inhibits the mitogenic responses of CD4^+^ T cells. Studies conducted to address the molecular mechanisms underlying these effects have shown that the impairment of TCR/CD3-mediated activation of CD4^+^ T cells by *T. cruzi*-derived mucins was correlated with induction of G1-phase cell cycle arrest. Importantly, it has been demonstrated that interactions of the terminal sialyl residues of *T. cruzi* mucins with CD4^+^ T cells led to the induction of the cell cycle regulator p27/Kip1 responsible to block the transition from G1 to S phase of mytosis, thus preventing the proliferative responses (Nunes et al., 2013).

Interestingly, the limited T cell responses observed in *T. cruzi* infection contrast with the large polyclonal expansion of B lymphocytes seen in the acute phase (Ortiz-Ortiz et al., 1980), as demonstrated by the increased frequency of IgG2a and IgG2b secreting B cells in peripheral lymphoid organs of infected mice. This phenomenon results in high frequency of nonspecific antibodies with low affinity for *T. cruzi* antigens (Ouaissi et al., 2001), some of them cross-reacting with heart and neural autoantigens (Acosta and Santos-Buch, 1985; Kierszenbaum, 1999; Engman and Leon, 2002). The auto-reactive B cell responses are thought to play secondary roles in the pathogenesis of Chagas disease. The extensive polyclonal expansion of the B cells could partly affect lymphoid compartments by increasing the competition for activation and survival signals needed to promote the generation of antigen-specific lymphocyte responses against *T. cruzi* (Freitas and Rocha, 2000; Montaudouin et al., 2013).

In addition, parasite-derived glycol-inositol-phospholipids (GIPLs), which are components of the dense glycolipid layer covering the parasite cell surface, also promote alterations in the B cell compartment. These molecules work as TLR4 agonists, mediating pro-inflammatory effects (Oliveira et al., 2004). Another virulence factor encoded by *T. cruzi* that target the B cell compartment is the proline racemase, which participates in arginine and proline metabolism, acting as a potent mitogen for B cells. Shortly, *T. cruzi* -derived virulence factors are active players in the subversion of the host immune system and are determinant for the establishment of chronic persistent infection (Reina-San-Martín et al., 2000; Chamond et al., 2003).

## The immune-endocrine imbalance is a key determinant of parasite persistence

Immune and neuro-endocrine systems are integrated through a complex network of mediators, involving cytokines, adipocytokines, hormones, and neuropeptides that collectively act to maintain homeostasis (Besedovsky and del Rey, 1996; Fantuzzi, 2005). However, when vertebrate hosts are challenged by infectious pathogens, acute and short-term stress signals are delivered by this network to initiate and build global host mechanisms of defense (Besedovsky and del Rey, 1996). In parallel, pathogens could interfere with this neuro-endocrine response at several levels. Thus, a race between pathogen-mediated evasion mechanisms and host immune response will determine whether the microorganisms will be rapidly eliminated or persist in the host (Figure 2D). In mice, the anti-*T. cruzi* immune response is associated with the activation of neuro-endocrine circuitries, mainly the hypothalamic-pituitary-adrenocortical (HPA) axis (Roggero et al., 2006; Corrêa-De-Santana et al., 2006b). In this scenario, pro-inflammatory cytokines released during infection activate the HPA axis, leading to production of glucocorticoids (GC), crucial for host survival. Evidently, the neuro-endocrine circutries initiates an anti-inflammatory response attempting to minimize the infection-induced collateral tissue damage. However its immunoregulatory effect ultimately favor the parasitism and establishment of persistent infection. Comparative studies between susceptible and resistant experimental mice models have indicated that the course of *T. cruzi* infection strongly depends on the appropriate timing and magnitude of the immune-endocrine response (Roggero et al., 2006). Susceptible animals succumb as consequence of increased inflammatory response poorly counteracted by the HPA axis, while resistant animals develop a more balanced immune-endocrine response that lead to the establishment of a chronic infection and mild pathology. Moreover, when GC signaling was abrogated by adrenalectomy or treatment with GC receptor antagonist RU486, the severity of infection increased dramatically as a result of an augmented inflammation-based immunopathology (Roggero et al., 2006; Pérez et al., 2007). These findings indicate that a delicate balance between the immune and endocrine systems play a role in the establishment of chronic infections. Additionally, the activation of HPA axis leads to secretion of other adrenal steroids, such as dehydroepiandrosterone (DHEA). In this regard, the increased vulnerability of *T. cruzi* infected young animals was associated with a high corticosterone/DHEA-sulfate ratio as compared to the adult counterparts (Pérez et al., 2011). Similarly, patients with severe chronic chagasic myocarditis also revealed a disruption in the activation of HPA axis as characterized by decreased concentrations of DHEA-sulfate and unbalanced cortisol/DHEA-sulfate ratio in comparison to asymptomatic or healthy individuals (Pérez et al., 2011). Overall, these findings reinforce the view that during *T. cruzi* human infection there are endocrine disturbances that might favor parasite persistence, thus influencing the disease pathology.

Moreover, pro-inflammatory cytokines associated to *T. cruzi* infection such as TNF-α, IL-6, or IL-1β could affect the release of hypothalamic, pituitary or adrenal hormones by their direct action on the endocrine glands (Kanczkowski et al., 2013, 2015; Hueston and Deak, 2014). During experimental *T. cruzi* infection, TNF-α has been implicated in the HPA activation at central level (Roggero et al., 2006; Pérez et al., 2007), although inhibitory actions at adrenal level has been also observed (Villar et al., 2013). Acutely infected TNF-R1 knock-out mice showed an enhanced transcription of adrenal steroidogenic proteins StAR, CYP11A1, CYP11B1 and 11β-HSD1 as compared to wild type mice, suggesting that GC secretion can be down regulated by TNF-α *in situ*, independently of the signaling pathway induced by adrenocorticotropic hormone (ACTH; Corrêa-De-Santana et al., 2006a; Villar et al., 2013). Since both parasites and their antigens had been detected within adrenal glands (Corrêa-De-Santana et al., 2006a; Villar et al., 2013), their presence might induce *in situ* the release of TNF-α, with the consequent modulation of GC secretion. In addition, IL-6 has also been associated with enhanced activity of the HPA axis during experimental *T. cruzi* infection. In this regard, supernatants of adenopituitary cell cultures challenged with the parasite contained more IL-6, while infected mice also showed augmented circulating levels of this cytokine systemically (Corrêa-De-Santana et al., 2006a). The activation of hypothalamus-pituitary unit also results in the release of both growth hormone (GH) and prolactin (PRL), which are capable of improving the immune response, counteracting the GC-driven immunosuppression. *T. cruzi* infection appears to directly modulate the secretion of both hormones, since *in vitro* infection of mammosomatotrophic cell line diminished GH and PRL secretion, similarly to observed in the pituitary glands of infected mice (Corrêa-De-Santana et al., 2009). The modulation of GH and PRL secretion by diminishing the Pit-1 gene expression, a major transcription factor for both hormone genes (Corrêa-De-Santana et al., 2009). Moreover, the downregulation of these hormones during the infection might be also related to the presence of parasites or their antigens in the glands, favoring T cell and macrophage infiltration, vascular stasis along with increased depots of extracellular matrix proteins (Corrêa-De-Santana et al., 2006b, 2009). The downmodulation of GH and PRL hormones is also observed in African trypanosomiasis and may illustrates a common modulatory mechanism (Radomski et al., 1994, 1996). Moreover, there is a bulk of evidence indicating that sex steroid hormones might influence the development and course of diverse parasitic infections (Romano et al., 2015). Particularly, it has been shown that *T. cruzi* parasites have the capacity to metabolize steroid hormones (Vacchina et al., 2008), suggesting a possible role of this mechanism in the host-parasite interplay.

## *Trypanosoma cruzi* infection may influence central tolerance

Several alterations in the thymic environment occur in infectious diseases (Watson et al., 1983, 1984; Savino et al., 1986; Leite de Moraes et al., 1991; Godfraind et al., 1995; Brito et al., 2003; Chen et al., 2005; Seixas and Ostler, 2005; Suzuki et al., 2005). The most evident alteration is the atrophy of the thymus due to the apoptotic death of differentiating thymocytes (Savino, 2006). In *T. cruzi* infected mice present a marked imbalance between intrathymic and systemic stress-related endocrine circuits, where the rise of intrathymic levels of GC affect the viability of double positive CD4^+^CD8^+^ (DP) cells, double-negative CD4^−^CD8^−^ (DN) and simple positive (SP) thymocytes (Roggero et al., 2006; Pérez et al., 2007). The induction of GC-driven apoptosis of DP cells is clearly associated with the activation of caspases 8 and 9 (Farias-de-Oliveira et al., 2013). In mice, the thymic atrophy is also influenced by the premature export of immature DP and DN thymocytes to the periphery, exhibiting a pro-inflammatory profile (Figure 2E; Leite-de-Moraes et al., 1992; De Meis et al., 2009; Morrot et al., 2011). Interestingly, increased numbers of circulating undifferentiated DP T lymphocytes was observed in patients with cardiac forms of chronic Chagas disease (Lepletier et al., 2014). Studies have identified a potential role for sphingosine-1-phosphate receptor-1 in this abnormal exit of undifferentiated thymocytes to the periphery in Chagas disease (Lepletier et al., 2014).

The *T. cruzi* infected thymus undergoing atrophy is still able to carry out negative selection, remaining important considerations in the context of host-pathogen interactions (Mendes-da-Cruz et al., 2003; Morrot et al., 2011). *T. cruzi* parasites also colonize the thymus (Savino et al., 1989), so their antigens may be presented to recirculating parasite-specific memory T cells migrating from the periphery to the thymic microenvironment. Alternatively, the parasite colonization of thymus could lead to the generation of *T. cruzi*-specific Tregs with high affinity TCR (Pacholczyk and Kern, 2008), thus promoting the host tolerance to persistent infection. Interestingly, it has been observed that chagasic patients in the indeterminate phase shown high frequencies of circulating Tregs as compared to chronic cardiac ones (De Araujo et al., 2011), suggesting a beneficial role of Tregs in suppressing the pathology associated to disease progression. In contrast, in experimental lethal models of Chagas disease with highly Th1-polarized inflammatory responses, the expansion of Tregs is clearly restricted (González et al., 2015).

## Concluding remarks

Recent studies suggest that the immuno-endocrine host response may favor *T. cruzi* chronic persistence. Future studies attempting to understand how *T. cruzi* evade host immune response or the extent by which parasite persistence might be favored by immune-neuro-endocrine regulation will provide important clues to better dissect mechanisms underlying the pathophysiology of Chagas disease.

## Author contributions

AM, FBG, SV and AP wrote the paper. All authors read and approved the final version of the manuscript.

### Conflict of interest statement

The authors declare that the research was conducted in the absence of any commercial or financial relationships that could be construed as a potential conflict of interest.
